# Pulmonary mycosis caused by *Papiliotrema laurentii* following COVID-19 infection: case report

**DOI:** 10.17843/rpmesp.2025.423.14970

**Published:** 2025-09-29

**Authors:** Josué M. Flores-Espejo, Roger A. Sernaqué-Mechato

**Affiliations:** 1 Facultad de Medicina Humana, Universidad de Piura, Lima, Peru. Universidad de Piura Facultad de Medicina Humana Universidad de Piura Lima Peru; 2 Departamento de Medicina Interna, Hospital Santa Rosa, Lima, Peru. Departamento de Medicina Interna Hospital Santa Rosa Lima Peru

**Keywords:** Papiliotrema laurentii, Lung Diseases, Fungal, COVID-19, SARS-CoV-2

## Abstract

*Papiliotrema laurentii* (formerly known as *Cryptococcus laurentii*) is an environmental saprophytic yeast that rarely causes disease in humans, mainly in immunosuppressed patients. We present a case of nosocomial pulmonary mycosis due to *Papiliotrema laurentii* in a 51-year-old male patient with a COVID-19 infection and a history of untreated type 2 diabetes mellitus. During his hospital stay, he received intravenous fluconazole for two weeks, with a favorable outcome. He was discharged with oral fluconazole for three months and follow-up in an outpatient clinic. One month after hospital discharge, he presented with cardiorespiratory arrest that did not respond to resuscitation maneuvers. The case highlights the importance of considering saprophytic species among the differential diagnoses of opportunistic mycoses.

## INTRODUCTION

*Papiliotrema laurentii* is a yeast-like basidiomycetous fungus that plays a saprophytic role in the environment, although in immunosuppressed patients, it can behave as an opportunistic pathogen. This species (formerly classified within the genus *Cryptococcus* spp.) accounted for 80% of cryptococcosis cases caused by saprophytic species ^1^. The Infectious Diseases Society of America (IDSA) has recognized it as a potentially pathogenic fungus, although it is rarely clinically relevant ^2^. Few cases of mycosis caused by this agent have been reported, with disseminated forms (fungemia) being more frequent than localized ones (cutaneous, ophthalmological, pulmonary, peritoneal, urinary, and central nervous system) ^3^. This article reports a case of nosocomial pulmonary mycosis by *Papiliotrema laurentii* in a patient at the Hospital Santa Rosa in Pueblo Libre, Lima (Peru).

## CASE REPORT

### Patient information

A 51-year-old male, from the district of Lince (an urban residential area in the department of Lima), with a history of type 2 diabetes mellitus and diabetic polyneuropathy, both without medical treatment. He was admitted to the hospital emergency department with hyperglycemia (454 mg/dL), type I respiratory failure (PaO2/FiO2 ratio of 278 mmHg), and a positive reverse transcriptase-polymerase chain reaction (RT-PCR) test for SARS-CoV-2.

By meeting the criteria for systemic inflammatory response syndrome (heart rate 95 beats/minute, respiratory rate 22 breaths/minute, and leukocytes 18,280/mm³ with lymphocytes 860/mm³), on his day of hospitalization (DOH) 2, he was admitted to the intensive care unit (ICU) with a diagnosis of severe COVID-19. Treatment was initiated with 2 g of intravenous ceftriaxone every 24 hours, 500 mg of azithromycin administered via nasogastric tube every 24 hours, and a sliding scale of regular insulin.

During his first stay in the ICU, laboratory tests showed albumin 2.6 g/dL and glycated hemoglobin 16.6%. On DOH 4, he was transferred to the Department of Internal Medicine. He returned to the ICU on DOH 7 after presenting a one-minute tonic-clonic seizure. An orotracheal tube was placed for mechanical ventilation due to a Glasgow Coma Scale of 7/15 and a respiratory rate of 6 breaths per minute. A non-contrast brain tomography was performed, which showed no lesions.

During his second stay in the ICU, he continuously used invasive devices: mechanical ventilation (two weeks with an orotracheal tube and two weeks with a tracheostomy tube), right radial arterial line, central venous catheter, peripheral venous catheter, and Foley catheter. On DOH 18, he developed ventilator-associated nosocomial pneumonia due to extended-spectrum β-lactamase-producing *Enterobacter aerogenes*. He received treatment with 1 g of intravenous meropenem every 8 hours for two weeks. In addition, he had negative results for tuberculosis (GeneXpert in sputum), syphilis (VDRL), viral hepatitis B (HBsAg and total anti-HBc), viral hepatitis C (anti-HCV), and human immunodeficiency virus (RT-PCR).

On DOH 38, he was transferred back to the Department of Internal Medicine as he was asymptomatic and on oxygen therapy via binasal cannula at 2 liters per minute. However, two days later (DOH 40), he again presented with respiratory symptoms.

### Clinical findings

On DOH 40, the patient (still with a tracheostomy tube) presented a febrile peak (39 °C), cough with yellowish-green expectoration, and dyspnea with increased oxygen requirements (high-flow cannula at 10 liters per minute). On physical examination, crackles were auscultated at the bases of both hemithoraces.

### Diagnostic approach

**Figure 1:**
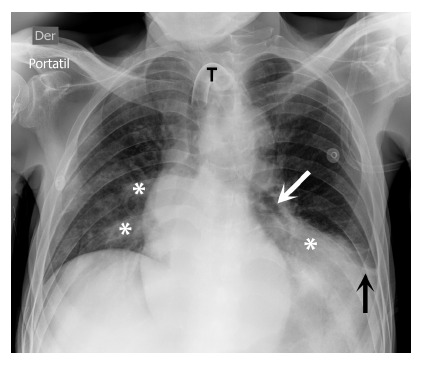
Diagnostic image of nosocomial pulmonary mycosis.

The same day the new respiratory symptoms began (DOH 40), a complete blood count was obtained showing leukocytosis and lymphopenia (Table 1), as well as a portable chest X-ray, which showed cotton-wool consolidations and left pleural effusion (Figure 1). A diagnosis of a second episode of nosocomial pneumonia was established. Blood and bronchial secretions were collected for culture. Given that the patient had previously received two antibiotic regimens (ceftriaxone with azithromycin and later meropenem), a nosocomial pulmonary mycosis was suspected.

**Table 1:** Serial control laboratory characteristics.

Laboratory test	DOH 40	DOH 41	DOH 45	DOH 50^a^	DOH 55	DOH 58^b^
Hemoglobin (mg/dL)	9,2	9,2	9,8	11,6	10,8	11,0
Leukocytes (cells/mm^3^)	12 930	10 680	9970	11 140	8170	8900
Neutrophils (cells/mm^3^)	11 507	8710	7760	7690	5780	6170
Lymphocytes (cells/mm^3^)	387	1190	1600	2780	1950	2020
Urea (mg/dL)	20	18	NR	NR	43	27
Serum creatinine (mg/dL)	0,26	0,25	NR	NR	0,39	0,30
Quantitative C-reactive protein (mg/dL)	13,30	NR	5,11	0,98	1,16	1,29

DOH: day of hospitalization, NR: test not performed.

a Laboratory control one week after the start of intravenous fluconazole.

b Laboratory control one day before the discharge summary.

Anteroposterior chest radiograph. A tracheostomy tube (T), bilateral cotton-wool consolidations (*), air bronchogram (white arrow), and blunting of the left costophrenic angle (black arrow) suggestive of pleural effusion are seen.

On DOH 43, the following microbiological results were reported: negative blood cultures and a positive bronchial secretion culture on Sabouraud agar. Microscopy, using Gram stain, showed 1+ pseudohyphae and more than 100 leukocytes per field (95% neutrophils). Through the VITEK® 2 automated system, only the species *Papiliotrema laurentii* was identified. No confirmatory molecular tests or antifungal susceptibility studies were performed.

### Therapeutic interventions

The same day *Papiliotrema laurentii* was identified (DOH 43), treatment was initiated with 200 mg of intravenous fluconazole every 12 hours for two weeks.

### Follow-up and outcomes

During the two weeks of intravenous fluconazole treatment, the patient showed good clinical and laboratory evolution (Table 1). Serial control blood cultures were negative, glycated hemoglobin decreased to 9.3%, and albumin increased to 3.0 g/dL.

**Figure 2:**
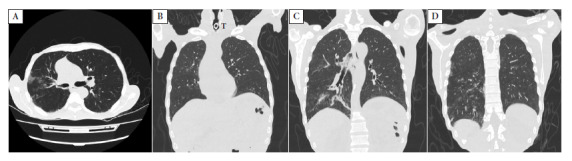
Imágenes de control al finalizar el tratamiento endovenoso.

At the end of this intravenous treatment (DOH 57), a control liver profile was performed, showing no significant alterations: aspartate aminotransferase 21 IU/L, alanine aminotransferase 25 IU/L, alkaline phosphatase 167 IU/L, gamma-glutamyl transferase 86 IU/L, total bilirubin 0.42 mg/dL, and direct bilirubin 0.19 mg/dL. In addition, a control non-contrast chest tomography was performed, which showed resolving lesions (Figure 2).

Non-contrast chest computed tomography in axial (image A) and coronal (from anterior to posterior: images B, C, and D) views. Image A: ground-glass opacity in the right upper lobe. Image B: tracheostomy tube (T), no parenchymal lesions are seen. Image C: residual consolidations with an air bronchogram in the right lower lobe. Image D: resolving subpleural inflammation in the right lower lobe; no left basal pleural effusion is evident.

Medical discharge occurred on DOH 59, with an indication for oral fluconazole 150 mg every 24 hours for three months, NPH insulin 5 IU subcutaneously every morning, and ambulatory follow-up in the outpatient clinic.

**Figure 3:**
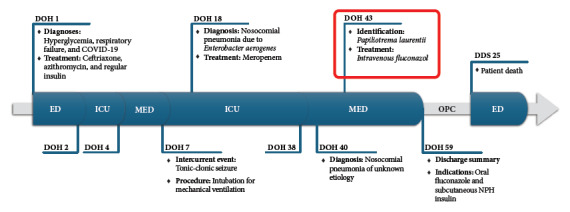
Timeline of the chronological report.

One month after the discharge summary, the patient was readmitted through the emergency department with signs of respiratory distress, tachypnea (22 breaths/minute), and an oxygen saturation of 98% with a binasal cannula at 2 liters per minute. The capillary glucose was 32 mg/dL. He was admitted to the Shock-Trauma Unit, where he suffered a cardiorespiratory arrest and died 25 minutes after the start of cardiopulmonary resuscitation maneuvers (Figure 3).

DOH: day of hospitalization, DDS: days since discharge summary, ED: emergency department, ICU: intensive care unit, MED: hospitalization area of the Department of Internal Medicine, OPC: monitoring by outpatient clinic.

## DISCUSSION

According to the Manual of Technical Procedures of the Microbiology Laboratory of the Hospital Santa Rosa, all received samples must be cultured on agars for bacteria and fungi. When the reported patient developed ventilator-associated pneumonia (DOH 18), the cultures were positive only for *Enterobacter aerogenes*; no fungi were identified on that occasion. Since *Papiliotrema laurentii* is not part of the normal lung microbiome ^4^, its subsequent isolation suggests that the patient acquired the microorganism within the hospital.

The presence of a clinical presentation compatible with the isolation of a germ allows distinguishing an infection (symptomatic patient) from colonization (asymptomatic patient) ^5^. The reported patient presented with fever, productive cough, dyspnea, increased oxygen requirements, and crackles in both hemithoraces. In addition, the chest X-ray showed signs of left lower lobe pneumonia with parapneumonic pleural effusion.

It has been documented that the prolonged use of invasive devices is the main risk factor for developing an infection by *Papiliotrema laurentii*^6^. Therefore, in a patient with respiratory symptoms, risk factors (carrier of a tracheostomy tube for 18 days until the onset of symptoms), and a positive sputum culture for *Papiliotrema laurentii* (without isolation of another pathogen), a pulmonary infection by this agent should be considered.

Given that the respiratory symptoms appeared 48 hours after the transfer from the ICU to the Internal Medicine service, it is difficult to determine in which hospital environment the infection was acquired. The respiratory tract is the main system affected in ICU-acquired infections (60.1%), and in 16.4% of positive cultures from these units, fungi are identified ^7^.

Recently, a model has been developed that evaluates whether nosocomial mycoses could have been acquired in the ICU. Using the Least Absolute Shrinkage and Selection Operator (LASSO) technique, the relevant variables identified were the duration of use of arterial catheter, urinary catheter, mechanical ventilation, broad-spectrum antibiotics, corticosteroids, and enteral nutrition. The model, built using Machine Learning and including these six variables, achieved a sensitivity of 96% and a specificity of 99% for determining if the mycoses were acquired in the ICU. In addition, the duration of arterial catheter and mechanical ventilation use were identified as independent risk factors for the initiation of antifungal therapy ^8^.

It is likely that the patient in the reported case acquired the *Papiliotrema laurentii* infection during his stay in the ICU. Of the variables considered in the model, during his second hospitalization in that unit, the patient used a right radial arterial catheter, assisted mechanical ventilation, a urinary catheter, and broad-spectrum antibiotics (meropenem). The first two variables have also been associated with the subsequent indication of antifungal treatment (intravenous fluconazole).

The diagnosis was made by culturing bronchial secretions on Sabouraud agar, with microscopy revealing the presence of 1+ pseudohyphae, as confirmed by the Gram stain. Cultures on media for bacteria were negative. The MALDI-TOF (matrix-assisted laser desorption/ionization time-of-flight) mass spectrometry technique with the VITEK 2 automated system was used for microorganism identification. This method uses spectra based on the protein profile, which is unique to a species, and allows for the correct identification of up to 95% of isolates ^9^.

However, in an epidemiological surveillance study conducted in China, 95.2% of isolates initially classified by the VITEK 2 system as *Papiliotrema laurentii* turned out to be erroneously classified. Sequencing of the internal transcribed spacer (ITS) revealed that these isolates actually corresponded to *Cryptococcus neoformans* (47.5%), *Candida* spp. (42.5%), *Arthrographis kalrae*, *Pseudozyma* spp., and *Sporobolomyces* spp. ^10^.

Therefore, one of the main limitations with the reported patient’s workup was the absence of molecular diagnostic tests (genomic sequencing) and antifungal susceptibility testing for the isolated strain. For example, in a case of fungemia by *Papiliotrema laurentii* reported in Brazil ^11^, the result obtained with the VITEK system was corroborated by sequencing the large subunit of ribosomal RNA and the ITS region. In addition, antifungal susceptibility analysis was performed, which showed a high minimum inhibitory concentration for fluconazole (4-8 μg/mL).

The patient in this report received the recommended treatment for mild cases of isolated pulmonary cryptococcosis: fluconazole 400 mg every 24 hours for three to twelve months ^12^. He showed good clinical evolution with intravenous treatment, was discharged with a resolving condition and follow-up in the outpatient clinic with an indication for oral fluconazole. A possible cause of his death was the inadequate management of insulin therapy in the out-of-hospital setting, as, despite having an indication for a single daily dose, the patient was readmitted with severe hypoglycemia.

In conclusion, this report highlights the importance of considering opportunistic mycoses as a differential diagnosis in cases of nosocomial pneumonia (even by uncommon pathogens like *Papiliotrema laurentii*), recognizing the risk factors associated with ICU-acquired infections, and using appropriate diagnostic techniques to promote timely identification, early initiation of treatment, and consequently, an improvement in the prognosis of these patients.
