# Supplementary material for: Pulmonary mycosis caused by Papiliotrema laurentii following COVID-19 infection: case report
Source: Rev Peru Med Exp Salud Publica. 2025 Sep 29;42(3):323–7. doi: 10.17843/rpmesp.2025.423.14970 (PMC12679970; doi:10.17843/rpmesp.2025.423.14970)
Supplement: Translation

# Translation: Micosis pulmonar por *Papiliotrema laurentii* posterior a infección por COVID-19: reporte de caso

**Authors:** Josué M. Flores-Espejo, Roger A. Sernaqué-Mechato

**Affiliations:** 1 Facultad de Medicina Humana, Universidad de Piura, Lima, Perú.; 2 Departamento de Medicina Interna, Hospital Santa Rosa, Lima, Perú.

**Keywords:** Papiliotrema laurentii, Enfermedades Pulmonares Fúngicas, COVID-19, SARS-CoV-2

## Abstract

*Papiliotrema laurentii* (anteriormente denominado *Cryptococcus laurentii*) es una levadura saprófita ambiental que rara vez causa enfermedad en humanos, principalmente en pacientes inmunosuprimidos. Se presenta un caso de micosis pulmonar nosocomial por *Papiliotrema laurentii* en un paciente varón de 51 años con infección por COVID-19 y antecedente de diabetes mellitus tipo 2 sin tratamiento. Durante su estancia hospitalaria recibió fluconazol endovenoso durante dos semanas, con evolución favorable. Fue dado de alta con fluconazol oral por tres meses y seguimiento en consulta externa. Un mes después del egreso hospitalario, presentó un paro cardiorrespiratorio que no respondió a maniobras de reanimación. El caso resalta la importancia de considerar a las especies saprófitas entre los diagnósticos diferenciales de micosis oportunistas.

## INTRODUCCIÓN

*Papiliotrema laurentii* es un hongo basidiomiceto levaduriforme que cumple un rol saprófito en el medio ambiente, aunque en pacientes inmunosuprimidos puede comportarse como patógeno oportunista. Esta especie (anteriormente clasificada dentro del género *Cryptococcus spp.*) formaba parte del 80% de casos de criptococosis causados por especies saprófitas ^1^. Incluso, la Sociedad de Enfermedades Infecciosas de América (IDSA, por sus siglas en inglés) lo ha reconocido como un hongo potencialmente patógeno, aunque rara vez clínicamente relevante ^2^.

Se han reportado pocos casos de micosis causadas por este agente, siendo más frecuente las formas diseminadas (fungemia) que las localizadas (cutáneas, oftalmológicas, pulmonares, peritoneales, urinarias y del sistema nervioso central) ^3^. El presente artículo reporta un caso de micosis pulmonar nosocomial por *Papiliotrema laurentii* en un paciente del Hospital Santa Rosa en Pueblo Libre, Lima (Perú).

## REPORTE DE CASO

### Información del paciente

Varón de 51 años, procedente del distrito de Lince (área urbana residencial del departamento de Lima), con antecedentes de diabetes mellitus tipo 2 y polineuropatía diabética, ambas sin tratamiento médico. Ingresó al servicio de emergencia hospitalaria con hiperglucemia (454 mg/dL), insuficiencia respiratoria tipo I (relación PaO_2_/FiO_2_ de 278 mmHg) y prueba de reacción en cadena de la polimerasa con transcriptasa inversa (RT-PCR, por sus siglas en inglés) positiva para SARS-CoV-2.

Por cumplir con criterios de síndrome de respuesta inflamatoria sistémica (frecuencia cardiaca 95 latidos/minuto, frecuencia respiratoria 22 respiraciones/minuto y leucocitos 18 280/mm^3^ con linfocitos 860/mm^3^), en su día de hospitalización (DDH) 2 fue admitido en la unidad de cuidados intensivos (UCI) con el diagnóstico de COVID-19 severo. Se indicó tratamiento con 2 g de ceftriaxona intravenosa cada 24 horas, 500 mg de azitromicina por sonda nasogástrica cada 24 horas e insulina R en escala móvil.

Durante su primera estancia en la UCI, los exámenes de laboratorio mostraron albúmina 2,6 g/dL y hemoglobina glicosilada 16,6%. En el DDH 4, fue trasladado al Departamento de Medicina Interna. Retornó a la UCI en el DDH 7 tras presentar una convulsión tónico-clónica de un minuto de duración. Se le colocó un tubo orotraqueal para ventilación mecánica debido a una escala de Glasgow de 7/15 y una frecuencia respiratoria de 6 respiraciones por minuto. Se realizó una tomografía cerebral sin contraste, la cual no evidenció lesiones.

Durante su segunda estancia en la UCI, utilizó de forma continua dispositivos invasivos: ventilación mecánica (dos semanas con tubo orotraqueal y dos semanas con tubo de traqueostomía), línea arterial radial derecha, catéter venoso central, catéter venoso periférico y sonda Foley. El DDH 18, desarrolló una neumonía nosocomial asociada a ventilación mecánica por *Enterobacter aerogenes* productora de β-lactamasas de espectro extendido. Recibio tratamiento con 1 g de meropenem endovenoso cada 8 horas durante dos semanas. Además, presentó resultados negativos para tuberculosis (GeneXpert en esputo), sífilis (VDRL), hepatitis viral B (HBsAg y anti-HBc total), hepatitis viral C (anti-VHC) y virus de inmunodeficiencia humana (RT-PCR).

En el DDH 38, fue trasladado nuevamente al Departamento de Medicina Interna por encontrarse asintomático y con oxigenoterapia por cánula binasal a 2 litros por minuto. No obstante, dos días después (DDH 40), volvió a presentar síntomas respiratorios.

### Hallazgos clínicos

En el DDH 40, el paciente (aún portador de tubo de traqueostomía) presentó un pico febril (39 °C), tos con expectoración amarillo-verdosa y disnea con incremento en los requerimientos de oxígeno (cánula de alto flujo a 10 litro por minuto). Al examen físico, se auscultaban crepitantes en las bases de ambos hemitórax.

### Abordaje diagnóstico

El mismo día del inicio de los nuevos síntomas respiratorios (DDH 40) se obtuvo un hemograma con leucocitosis y linfopenia (Tabla 1), así como una radiografía portátil de tórax, en la que se observaron consolidaciones algodonosas y derrame pleural izquierdo (Figura 1). Se estableció el diagnóstico de un segundo episodio de neumonía nosocomial. Se recolectaron muestras de sangre y secreción bronquial para cultivos. Dado que el paciente había recibido previamente dos esquemas antibióticos (ceftriaxona con azitromicina y posteriormente meropenem), se sospechó una micosis pulmonar nosocomial.

**Tabla 1:** Características laboratoriales seriadas de control.

Examen auxiliar	DDH 40	DDH 41	DDH 45	DDH 50^a^	DDH 55	DDH 58^b^
Hemoglobina (mg/dL)	9,2	9,2	9,8	11,6	10,8	11,0
Leucocitos (cél/mm^3^)	12 930	10 680	9970	11 140	8170	8900
Neutrófilos (cél/mm^3^)	11 507	8710	7760	7690	5780	6170
Linfocitos (cél/mm^3^)	387	1190	1600	2780	1950	2020
Urea (mg/dL)	20	18	NR	NR	43	27
Creatinina sérica (mg/dL)	0,26	0,25	NR	NR	0,39	0,30
Proteína C reactiva cuantitativa (mg/dL)	13,30	NR	5,11	0,98	1,16	1,29

DDH: día de hospitalización, NR: prueba no realizada.

a Control laboratorial una semana posterior al inicio de fluconazol endovenoso.

b Control laboratorial un día anterior a la epicrisis.

**Figura 1:** Imagen del diagnóstico de micosis pulmonar nosocomial.

Radiografía de tórax en incidencia anteroposterior. Se aprecia tubo de traqueostomía (T), consolidaciones algodonosas bilaterales (*), broncograma aéreo (flecha blanca) y borramiento del ángulo costofrénico izquierdo (flecha negra) sugerente de efusión pleural.

En el DDH 43, se reportaron los siguientes resultados microbiológicos: hemocultivos negativos y cultivo de secreción bronquial en agar Sabouraud positivo. En la microscopía, se observaron pseudohifas 1+ y más de 100 leucocitos por campo (95% de neutrófilos), mediante tinción de Gram. A través del sistema automatizado VITEK® 2, se identificó únicamente la especie *Papiliotrema laurentii*. No se realizaron pruebas moleculares de confirmación ni estudios de sensibilidad a antifúngicos.

### Intervenciones terapéuticas

El mismo día que se identificó *Papiliotrema laurentii* (DDH 43), se indicó tratamiento con 200 mg de fluconazol endovenoso cada 12 horas por dos semanas.

### Seguimiento y resultados

Durante las dos semanas de tratamiento con fluconazol endovenoso, el paciente presentó buena evolución clínica y laboratorial (Tabla 1). Los hemocultivos seriados de control resultaron negativos, la hemoglobina glicosilada disminuyó a 9,3% y la albúmina aumentó a 3,0 g/dL.

Al finalizar este tratamiento endovenoso (DDH 57), se realizó un perfil hepático de control, sin alteraciones significativas: aspartato aminotransferasa 21 UI/L, alanina aminotransferasa 25 UI/L, fosfatasa alcalina 167 UI/L, gamma glutamil transferasa 86 UI/L, bilirrubina total 0,42 mg/dL y bilirrubina directa 0,19 mg/dL. Además, se efectuó una tomografía de tórax sin contraste de control, en la que se observaron lesiones en resolución (Figura 2).

**Figura 2:** Imágenes de control al finalizar el tratamiento endovenoso.

Tomografía de tórax sin contraste en cortes axial (imagen A) y coronal (de anterior a posterior: imágenes B, C y D). Imagen A: patrón de vidrio deslustrado en lóbulo superior derecho. Imagen B: tubo de traqueostomía (T), no se evidencia lesiones en el parénquima pulmonar. Imagen C: consolidaciones residuales con broncograma aéreo en lóbulo inferior derecho. Imagen D: inflamación subpleural en resolución en lóbulo inferior derecho, no se evidencia derrame pleural basal izquierdo.

El alta médica se dio en el DDH 59, con indicación de fluconazol 150 mg de por vía oral cada 24 horas por tres meses, insulina NPH 5 UI subcutáneas cada mañana y seguimiento ambulatorio por consulta externa.

Un mes posterior de la epicrisis, el paciente reingresó por el servicio de emergencia con signos de dificultad respiratoria, taquipnea (22 respiraciones/minuto) y saturación de oxígeno de 98% con cánula binasal a 2 litros por minuto. La glucosa capilar fue de 32 mg/dL. Fue ingresado a la Unidad de Shock-Trauma, donde presentó un paro cardiorrespiratorio y falleció 25 minutos después del inicio de las maniobras de reanimación cardiopulmonar (Figura 3).

**Figura 3:** Línea de tiempo del relato cronológico.

DDH: día de hospitalización, DDE: días desde la epicrisis, EMG: emergencia, UCI: unidad de cuidados intensivos, MED: área de hospitalización del Departamento de Medicina Interna, CEX: monitoreo por consulta externa.

## DISCUSIÓN

Según el Manual de Procedimientos Técnicos del Laboratorio de Microbiología del Hospital Santa Rosa, todas las muestras recibidas deben ser cultivadas en agares para bacterias y hongos. Cuando el paciente reportado desarrolló la neumonía asociada a ventilación mecánica (DDH 18), los cultivos fueron positivos únicamente para *Enterobacter aerogenes*; en esa ocasión no se identificaron hongos. Dado que *Papiliotrema laurentii* no forma parte del microbioma pulmonar habitual ^4^, su posterior aislamiento sugiere que el paciente adquirió el microorganismo dentro del hospital.

La presencia de un cuadro clínico compatible con el aislamiento de un germen permite distinguir una infección (paciente sintomático) de una colonización (paciente asintomático)^5^. El paciente reportado presentó fiebre, tos productiva, disnea, incremento en los requerimientos de oxígeno y crepitantes en ambos hemitórax. Además, la radiografía de tórax evidenció signos de neumonía lobar inferior izquierda con efusión pleural paraneumónica.

Se ha documentado que el uso prolongado de dispositivos invasivos constituye el principal factor de riesgo para desarrollar una infección por *Papiliotrema laurentii*^6^. Por tanto, en un paciente con síntomas respiratorios, factores de riesgo (portador de tubo de traqueostomía durante 18 días hasta el inicio de los síntomas) y cultivo de esputo positivo para *Papiliotrema laurentii* (sin aislamiento de otro patógeno), debe considerarse una infección pulmonar por este agente.

Dado que los síntomas respiratorios aparecieron a las 48 horas después del traslado desde la UCI hacia el servicio de Medicina Interna, resulta difícil determinar en qué ambiente hospitalario se adquirió la infección. El tracto respiratorio es el principal sistema afectado en las infecciones adquiridas en UCI (60,1 %), y en el 16,4 % de los cultivos positivos provenientes de estas unidades se identifican hongos ^7^.

Recientemente, se ha desarrollado un modelo que evalúa si las micosis nosocomiales podrían haber sido adquiridas en la UCI. Mediante la técnica *Least Absolute Shrinkage and Selection Operator* (*LASSO*) se identificaron como variables relevantes los tiempos de uso de catéter arterial, catéter urinario, ventilación mecánica, antibióticos de amplio espectro, corticoides y nutrición enteral. El modelo construido mediante *Machine Learning* e incluyendo estas seis variables, obtuvo una sensibilidad del 96% y especificidad del 99% para determinar si las micosis fueron adquiridas en la UCI. Además, se identificó a los tiempos de uso de catéter arterial y de ventilación mecánica, como factores de riesgo independientes para el inicio de terapia antifúngica ^8^.

Es probable que el paciente del caso reportado haya adquirido la infección por *Papiliotrema laurentii* durante su estancia en la UCI. De las variables consideradas en el modelo, durante su segunda hospitalización en dicha unidad, el paciente utilizó catéter arterial radial derecho, ventilación mecánica asistida, catéter urinario y antibióticos de amplio espectro (meropenem). Las dos primeras variables también se han asociado con la posterior indicación de tratamiento antifúngico (fluconazol endovenoso).

El diagnóstico se realizó mediante cultivo de secreción bronquial en agar Sabouraud, cuya microscopía reveló la presencia de pseudohifas 1+ con la tinción de Gram. Los cultivos en medios para bacterias fueron negativos. Se empleó la técnica de espectrometría de masas MALDI-TOF (*matrix-assisted laser desorption/ionization time-of-flight*) con el sistema automatizado VITEK 2 para la identificación del microorganismo. Este método utiliza espectros basados en el perfil proteico, único para una especie, y permite identificar correctamente hasta un 95 % de los aislamientos ^9^.

No obstante, en un estudio de vigilancia epidemiológica realizado en China, el 95,2% de aislamientos inicialmente catalogados por el sistema VITEK 2 como *Papiliotrema laurentii* resultaron estar erróneamente clasificados. El secuenciamiento del espaciador transcrito interno (ITS, por sus siglas en inglés) reveló que dichos aislamientos realmente correspondían a *Cryptococcus neoformans* (47,5%), *Candida* spp*.* (42,5%), *Arthrographis kalrae*, *Pseudozyma* spp*.* y *Sporobolomyces* spp*.*^10^*.*

Por ello, una de las principales limitaciones con el plan de trabajo del paciente reportado fue la ausencia de pruebas de diagnóstico molecular (secuenciamiento genómico) y de sensibilidad a antifúngicos para la cepa aislada. Por ejemplo, en un caso de fungemia por *Papiliotrema laurentii* reportado en Brasil ^11^, el resultado obtenido con el sistema VITEK fue corroborado mediante secuenciamiento de la subunidad mayor de ARN ribosomal y de la región ITS. Además, se realizó análisis de sensibilidad a antifúngicos donde se observó una concentración inhibitoria mínima elevada para el fluconazol (4-8 μg/mL).

El paciente del presente reporte recibió tratamiento recomendado para cuadros leves de criptococosis pulmonar aislada: fluconazol 400 mg cada 24 horas durante tres a doce meses ^12^. Mostró buena evolución clínica con el tratamiento endovenoso, fue dado de alta con un cuadro en resolución y seguimiento por consultorio externo con indicación de fluconazol por vía oral. Una posible causa de su fallecimiento fue el manejo inadecuado de la insulinoterapia en el ámbito extrahospitalario, ya que, pese a tener indicación de dosis única diaria, el paciente reingresó con hipoglucemia severa.

En conclusión, este reporte destaca la importancia de considerar las micosis oportunistas como diagnostico diferencial en casos de neumonía nosocomial (aun por patógenos poco comunes como *Papiliotrema laurentii*), reconocer los factores de riesgo asociados a infecciones adquiridas en la UCI, y emplear técnicas diagnósticas apropiadas para favorecer una identificación oportuna, el inicio precoz del tratamiento y, en consecuencia, una mejora en el pronóstico de estos pacientes.
